# Color Doppler Ultrasound Versus Magnetic Resonance Imaging for Diagnosing Giant Cell Arteritis: A Systematic Review and Meta-Analysis

**DOI:** 10.7759/cureus.101096

**Published:** 2026-01-08

**Authors:** Mustapha El Yaman, Fatima Khan, Bareq S Al-Lami, Abdulrahman O Saeed, Baqir S Al-Lami, Yasir Al-Lami, Leen El Yaman

**Affiliations:** 1 Medicine, Conquest Hospital, East Sussex Healthcare NHS Trust, St Leonards-on-Sea, GBR; 2 Medicine, Royal Surrey County Hospital, Surrey, GBR; 3 Radiology, Royal Sussex County Hospital, Brighton, GBR; 4 Medicine, Hawler Medical University, Erbil, IRQ; 5 General Medicine, Hawler Medical University, Erbil, IRQ; 6 Internal Medicine, Northwick Park Hospital, London, GBR

**Keywords:** color doppler ultrasound, diagnostic accuracy, giant cell arteritis, magnetic resonance imaging, mri, ultrasound, vessel-wall imaging

## Abstract

Rapid diagnosis of giant cell arteritis (GCA) is essential to prevent ischemic complications. Color Doppler ultrasound (CDUS) and high-resolution magnetic resonance imaging (MRI) are increasingly used as alternatives or adjuncts to temporal artery biopsy, but their comparative diagnostic performance remains uncertain.

We performed a systematic review and bivariate random-effects meta-analysis of diagnostic accuracy studies in adults with suspected GCA. Studies reporting extractable 2×2 data against temporal artery biopsy, or accepted clinical reference standards when biopsy was unavailable, were included. Risk of bias was assessed using Quality Assessment of Diagnostic Accuracy Studies 2 (QUADAS-2). Pooled sensitivity, specificity, diagnostic odds ratios (DOR), and summary receiver operating characteristic (ROC) curves were calculated, with evaluation of heterogeneity and publication bias.

Thirty-nine studies, including 3,619 patients, met the inclusion criteria. Thirty-one studies assessed CDUS (2,766 patients) and 12 evaluated MRI (853 patients). For CDUS, the median sensitivity was 0.83 (range 0.17-1.00) and the median specificity was 0.88 (range 0.59-1.00), with a median DOR of 24.9 and substantial between-study variability. MRI demonstrated a median sensitivity of 0.88 (range 0.61-1.00), a median specificity of 0.92 (range 0.71-1.00), and a higher median DOR of 72.0, with more consistent estimates. Evidence of small-study or publication bias was observed for MRI (p≈0.0004) and was borderline for CDUS (p≈0.055). QUADAS-2 assessments were generally favorable, though common limitations included variable blinding, heterogeneous imaging protocols, and differences in corticosteroid timing.

MRI demonstrates higher and more consistent diagnostic performance than CDUS. CDUS can achieve high accuracy in experienced centers but shows notable operator dependence. Both modalities support imaging-based diagnostic pathways for GCA, with the choice influenced by local expertise, resource availability, and corticosteroid exposure.

## Introduction and background

Giant cell arteritis (GCA) is a vasculitis of medium- and large-sized arteries that mostly affects older adults and carries an immediate risk of irreversible visual loss and other ischemic complications. In practice, the risk of irreversible visual loss often leads to immediate initiation of high-dose corticosteroids prior to imaging or temporal artery biopsy (TAB), and corticosteroid exposure before testing can reduce the sensitivity of subsequent diagnostic investigations [1,2]. TAB remains a widely used reference standard and is highly specific when positive, but it is invasive and imperfect: segmental inflammation produces skip lesions and biopsies, and therefore, can be falsely negative even when disease is present, a limitation that has important consequences for both diagnosis and management [3,4].

Because of these shortcomings, noninvasive vascular imaging has become central to modern diagnostic pathways. Color Doppler ultrasound (CDUS) can demonstrate the hypoechoic circumferential “halo sign,” a marker of mural edema and probable active arteritis, and early work suggested the halo sign had good specificity in the appropriate clinical context [5,6]. High-resolution, contrast-enhanced magnetic resonance vessel-wall imaging also identifies concentric wall thickening and post-contrast mural enhancement that correlate with histologic inflammation of the temporal artery, and single-center and multicenter series have reported promising sensitivity and specificity for dedicated protocols [7]. Practice guidelines have moved to reflect these data: international task forces now recommend early imaging; ultrasound or MRI, as part of the diagnostic workup for suspected cranial GCA where local expertise and equipment permit, although uptake and recommended sequencing vary by region and resource availability [8]. Reported accuracy for both CDUS and high-resolution MRI varies substantially between studies, driven by differences in operator experience, scanner and probe parameters, study populations, steroid timing, and choice of reference standard.

The clinical question therefore remains unsettled: is one imaging test consistently accurate enough to be preferred as the primary noninvasive diagnostic alternative to biopsy, or do trade-offs in availability, reproducibility, and diagnostic performance mean both tests retain complementary roles? This review addresses that question by pooling and directly comparing diagnostic accuracy estimates for CDUS versus high-resolution MRI, while paying close attention to bias risks that commonly inflate test performance in single studies.

## Review

Methodology

We conducted a systematic review and meta-analysis of diagnostic accuracy studies of imaging for suspected GCA. Eligible studies enrolled adults with clinical features suggestive of GCA (for example, new temporal headache, jaw claudication, or acute visual symptoms). Studies were excluded if patients received high-dose corticosteroids for more than two weeks before imaging. Index tests evaluated were: (A) CDUS and (B) MRI of arteries relevant to GCA. For each study, we recorded the specific CDUS criteria used (for example, presence of a halo sign, intima-media thickness thresholds, unilateral versus bilateral criteria), probe frequency, arterial territories examined (temporal only versus temporal + axillary/extra-cranial), and for MRI the pulse sequences, use of dedicated head coils, field strength, contrast protocol, and the vessel-wall criteria applied.

Reference Standard and Verification

The primary reference standard was the temporal artery biopsy (histological demonstration of vasculitis). When a biopsy was not performed or reported, a study-level clinical diagnosis based on accepted criteria and clinical adjudication (commonly using American College of Rheumatology/European League Against Rheumatism (ACR/EULAR) clinical classification and local diagnostic pathways) [9] was accepted as a secondary reference standard. To address the potential for differential verification bias introduced by mixed reference standards, we prespecified subgroup and sensitivity analyses separating studies using biopsy alone from those using a clinical diagnosis (or composite reference) and performed analyses restricted to biopsy-confirmed cohorts where data permitted.

Data Sources and Search Strategy

We searched PubMed, Embase, Cochrane CENTRAL, and Google Scholar from inception to the date of the search. Search terms combined GCA vocabulary (e.g., “giant cell arteritis”, “temporal arteritis”) with imaging terms (“ultrasound”, “Doppler”, “halo”, “MRI”, “vessel wall enhancement”) and diagnostic keywords (“sensitivity”, “specificity”, “biopsy”). We supplemented database searches by screening reference lists of included studies and relevant reviews.

Study Selection and Data Extraction

Two reviewers independently screened titles/abstracts and full texts and extracted study data using a standardized form. For each imaging modality and study, we extracted or reconstructed 2×2 contingency tables (true positives, false negatives, true negatives, false positives) against the prespecified reference standard. When studies reported raw counts these were used directly. When only sensitivity, specificity and subgroup totals were provided, we back-calculated integer 2×2 counts by applying reported rates to reported denominators and rounding to the nearest integer; the specific rounding rule used was to round fractions ≥0.5 up and <0.5 down. We also extracted study design and conduct features relevant to heterogeneity and bias: study design (prospective/retrospective), sampling (consecutive/enriched sampling), patient demographics, clinical setting (fast-track/secondary/tertiary care), blinding of index test readers to reference standard results, timing between imaging and biopsy, and details of operator experience (sonographer training, radiologist expertise) and equipment where reported.

Risk of Bias Assessment

Each study was assessed with Quality Assessment of Diagnostic Accuracy Studies 2 tool (QUADAS-2) [10]. Domain-level judgements (patient selection, index test, reference standard, flow and timing) were completed independently by two reviewers, and disagreements were resolved by consensus. We reported QUADAS-2 domain ratings and used these to inform sensitivity analyses (for example, excluding studies at high risk in key domains).

Statistical Analysis and Investigation of Heterogeneity

We pooled sensitivity and specificity for each modality using a bivariate random-effects meta-analytic model [11]. We calculated pooled diagnostic odds ratios (DOR) and plotted summary receiver operating characteristic (SROC) curves. Heterogeneity was quantified using I² for sensitivity, specificity and DOR estimates, and visually inspected via forest and SROC plots.

Given the recognized influence of corticosteroids on the imaging yield, we extracted timing between steroid initiation and imaging (and between imaging and biopsy) whenever reported. Where possible we categorized imaging timing relative to steroid start (imaging before steroid initiation; imaging within zero to three days after initiation; imaging greater than three days after initiation) and included timing as a covariate in meta-regression or performed subgroup analyses. Where timing data were incompletely reported, we documented this and addressed it in sensitivity analyses or in explicit limitations.

Publication bias and small-study effects were assessed using funnel plots and Egger's regression tests as appropriate for diagnostic accuracy meta-analysis. All statistical analyses were performed using R programming meta-analysis software (packages for diagnostic meta-analysis and meta-regression) (Version 4.5.2, Foundation for Statistical Computing, Vienna, Austria, https://www.R-project.org/) and followed Preferred Reporting Items for a Systematic Review and Meta-analysis of Diagnostic Test Accuracy Studies (PRISMA-DTA) [12] reporting guidance. Results for the number of reconstructed tables, subgroup analyses, and sensitivity analyses are reported in the results section.

Results

**Figure 1:**
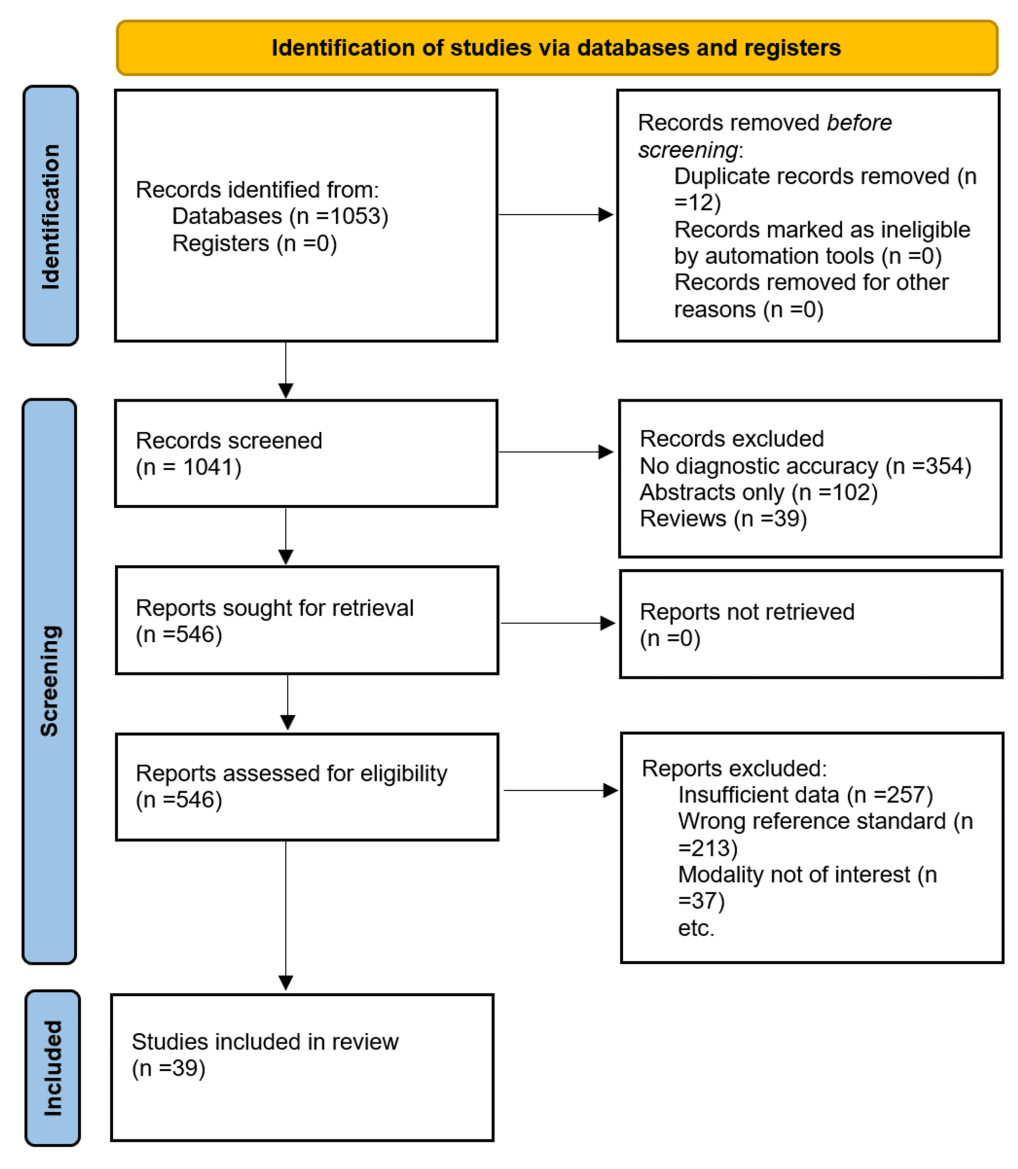
PRISMA flowchart PRISMA: Preferred Reporting Items for a Systematic Review and Meta-analyses.

Our systematic search and review process yielded a final set of 39 diagnostic accuracy studies [13-51], as shown in the PRISMA flow chart (Figure 1), meeting the inclusion criteria.

Of these, 31 studies evaluated CDUS and 12 evaluated MRI. The combined analysis includes data from a total of 3,619 patients with suspected GCA. The populations were predominantly drawn from specialized vascular or rheumatology clinics, reflecting a high pre-test probability of disease in most cohorts. Study characteristics are shown in Table 1.

**Table 1:** Characteristics of the included studies US: Ultrasound; TAB: temporal artery biopsy; GCA: giant cell arteritis.

First Author	Year	Design	Sample size
Luqmani et al. [13]	2016	Prospective, multicenter diagnostic cohort study	381
Hansen et al. [14]	2022	Prospective diagnostic accuracy cohort study	106 Analyzed (US + TAB available): 78
Aranda-Valera et al. [15]	2017	Prospective diagnostic accuracy cohort	451 suspected GCA TAB performed: 166 Final GCA diagnosis (clinical): 256
Molina-Collada et al. [16]	2022	Retrospective diagnostic accuracy cohort	198
Lecler et al. [17]	2022	Prospective single-center diagnostic accuracy cohort	45
Rodriguez-Régent et al. [44]	2020	Prospective diagnostic accuracy cohort	32
Bley et al. [18]	2008	Retrospective diagnostic-accuracy cohort	59
van Nieuwland et al. [19]	2025	Nested case–control diagnostic-accuracy study	42
Suelves et al. [20]	2010	Prospective pilot diagnostic-accuracy study	10
He et al. [21]	2022	Single-center retrospective diagnostic accuracy cohort study	63
Ghinoi et al. [22]	2008	Prospective diagnostic accuracy cohort study	20
Bley et al. [45]	2005	Prospective diagnostic accuracy cohort study	20
Croft et al. [23]	2015	Retrospective diagnostic cohort study	24
El-Jade et al. [24]	2024	Retrospective diagnostic accuracy cohort	38
Czihal et al. [25]	2020	Prospective diagnostic accuracy cohort	114
Black et al. [26]	2013	Retrospective diagnostic accuracy cohort	50 (TAB subset: 21)
Schmidt et al. [27]	1997	Prospective diagnostic accuracy cohort	30
Conway et al. [28]	2019	Prospective diagnostic accuracy cohort	162
LeSar et al. [29]	2002	Prospective diagnostic accuracy cohort	32
Kaandorp et al. [30]	2024	Prospective diagnostic accuracy cohort	242
Sundholm et al. [31]	2019	Prospective diagnostic accuracy cohort	75
Roncato et al. [32]	2017	Retrospective diagnostic accuracy cohort	42
Pérez-López et al. [33]	2009	Prospective diagnostic accuracy cohort	54
Maldini et al. [34]	2010	Retrospective diagnostic accuracy cohort	77
Romera-Villegas et al. [35]	2004	Prospective diagnostic accuracy cohort	68
Bilyk et al. [36]	2017	Prospective diagnostic accuracy cohort, masked	71
Nesher et al. [37]	2002	Prospective diagnostic accuracy cohort	69
Habib et al. [38]	2011	Prospective diagnostic accuracy cohort	32
Pfadenhauer et al. [39]	2003	Prospective diagnostic accuracy cohort	67
Murgatroyd et al. [40]	2003	Prospective diagnostic accuracy pilot study	26
Reinhard et al. [41]	2003	Prospective diagnostic accuracy cohort	83
Skoog et al. [42]	2024	Retrospective diagnostic accuracy cohort study	107
Aschwanden et al. [43]	2010	Prospective observational diagnostic study	72
Sommer et al. [46]	2018	Prospective monocentric cohort	27
Mohammad-Brahim et al. [47]	2018	Prospective single-center study	27
Rheuaume et al. [48]	2016	Prospective cohort study	171
Mournet et al. [49]	2021	Retrospective single-center	64
Junek et al. [50]	2021	Retrospective cohort	268
Klink et al. [51]	2014	Prospective multicenter cohort	185

Across the 39 included studies, most evaluated a single imaging modality, with only a small subset assessing both CDUS and MRI within the same patient cohort. As a result, pooled diagnostic accuracy estimates for CDUS and MRI were derived predominantly from separate study populations. Comparisons between modalities in this meta-analysis therefore represent indirect, study-level comparisons based on pooled sensitivity, specificity, and diagnostic odds ratios, rather than paired within-patient analyses.

**Figure 2:**
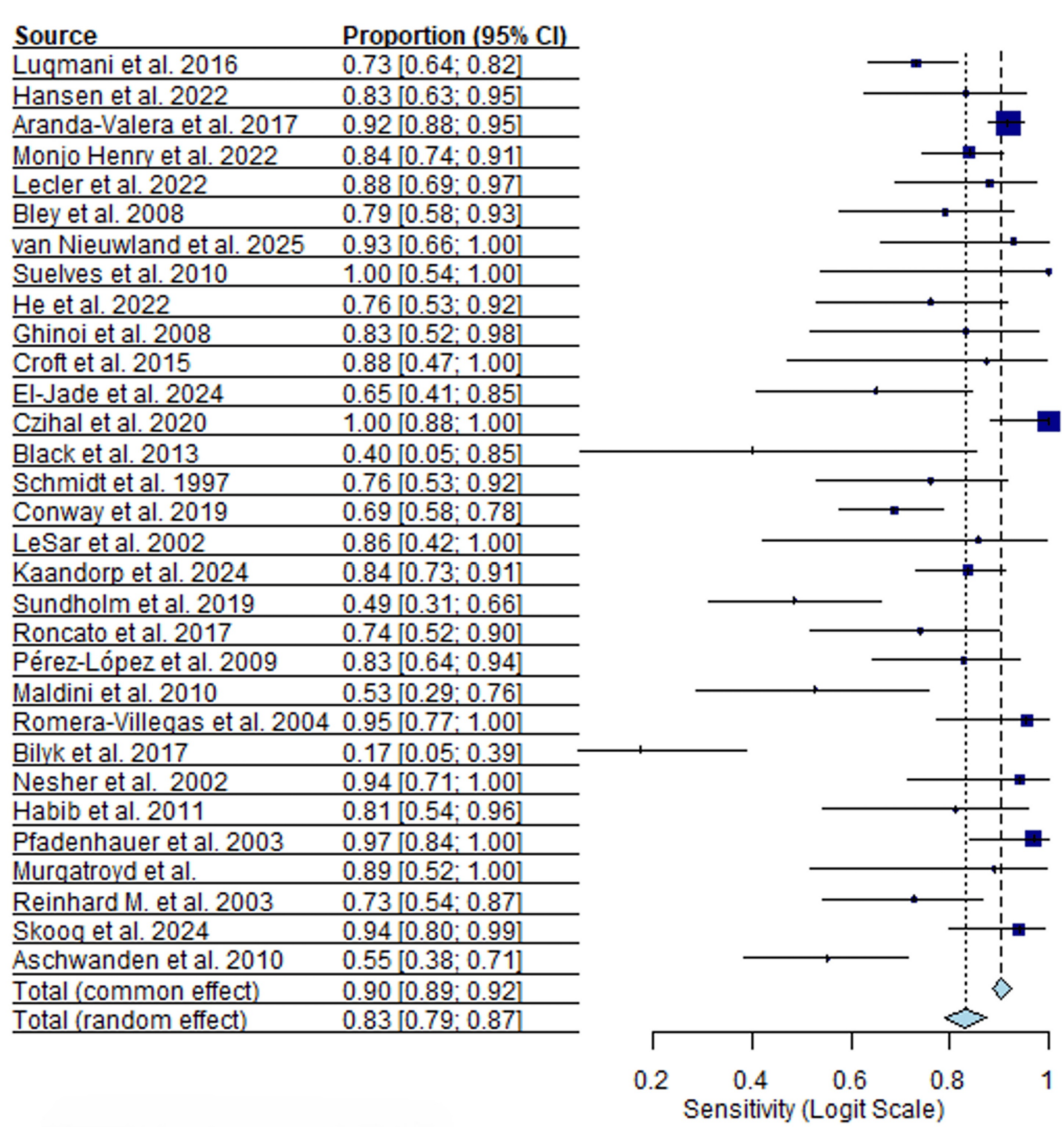
Ultrasound sensitivity forest plot [13-43]

**Figure 3:**
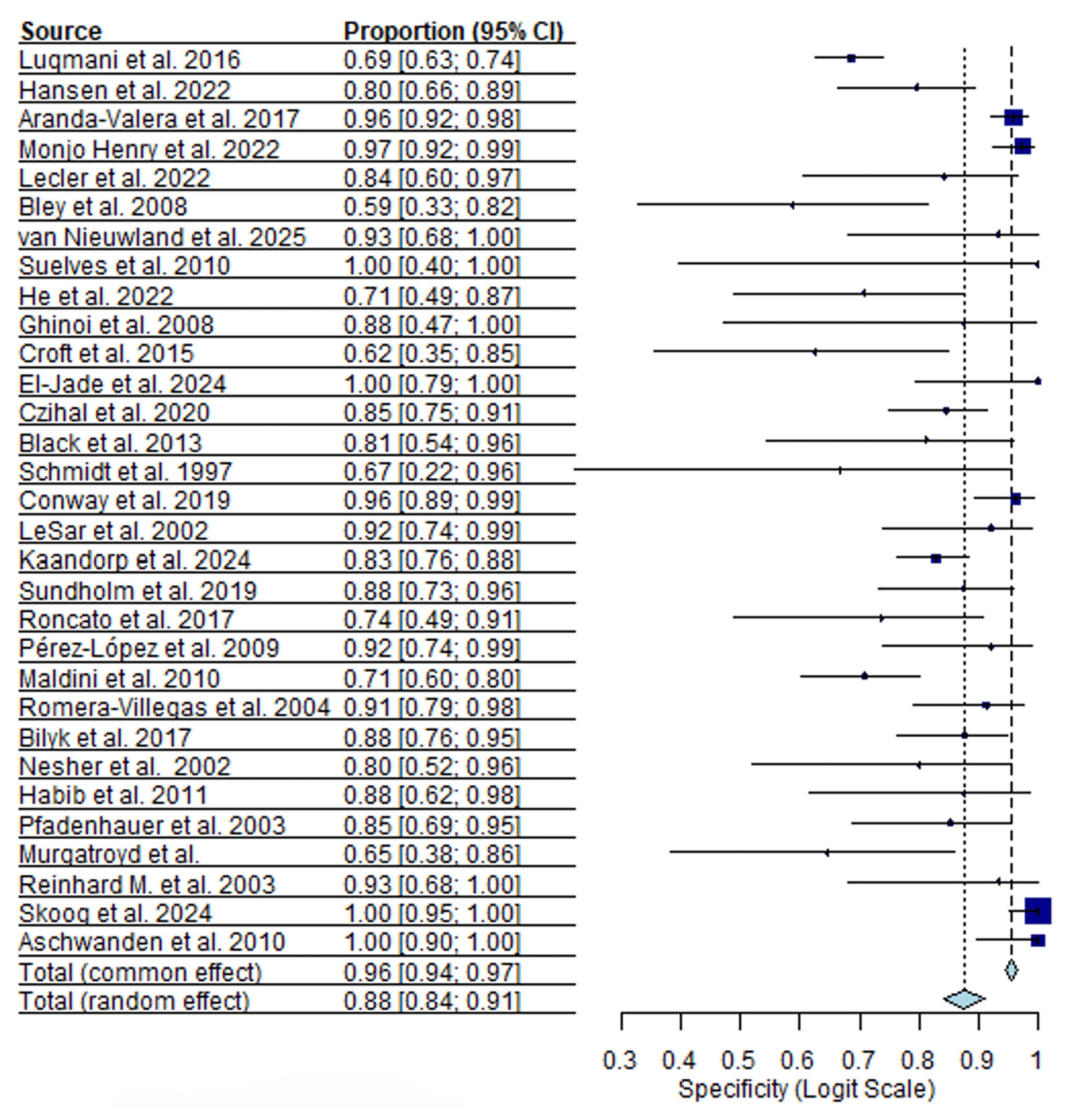
Ultrasound specificity forest plot [13-43]

Thirty-one ultrasound studies comprising 2,766 patients were included in the quantitative synthesis. Reported sensitivity values varied widely across studies, with a median sensitivity of 0.83 (95% CI, I²=51.7%) and a range from 0.17 to 1.00. Specificity estimates were more consistent but remained heterogeneous, with a median specificity of 0.88 (95% CI, I²=47.4%) and a range from 0.59 to 1.00. Forest plots for pooled sensitivity and specificity are shown in Figures 2, 3.

Moderate heterogeneity was observed across both diagnostic parameters for the ultrasound modality. 

**Figure 4:**
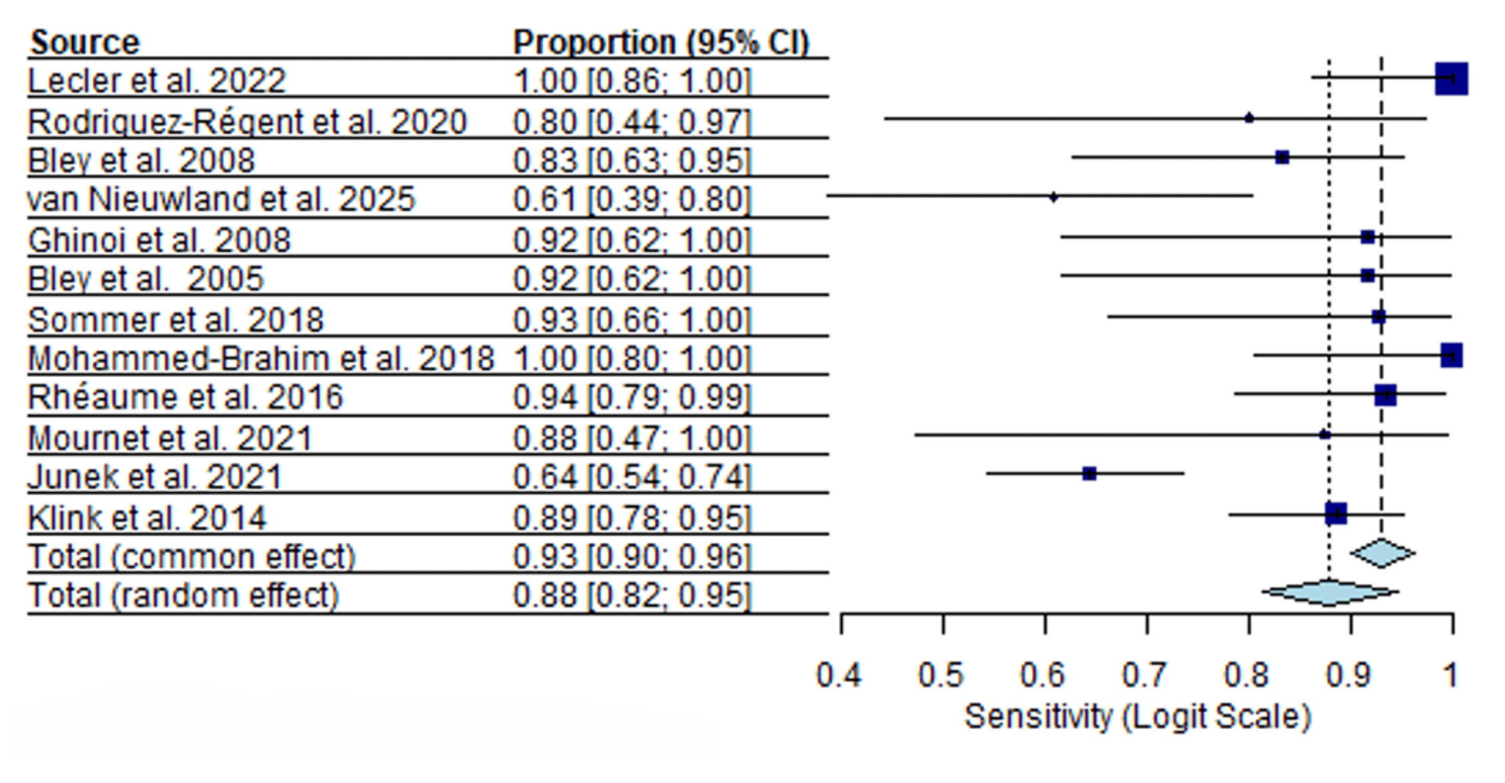
MRI sensitivity forest plot [17-19], [22], [44-51].

**Figure 5:**
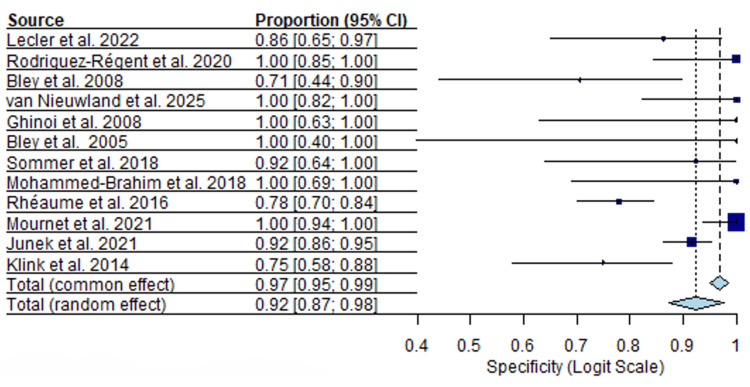
MRI specificity forest plot [17-19], [22], [44-51].

Twelve MRI studies including 853 patients were analyzed. Sensitivity estimates were generally high, with a median sensitivity of 0.88 (95% CI, I²=53.1%) and a range from 0.61 to 1.00. Specificity estimates showed a median of 0.92 (95% CI, I²=59.2%), ranging from 0.71 to 1.00. Forest plots for MRI sensitivity and specificity are presented in Figures 4, 5. 

**Figure 6:**
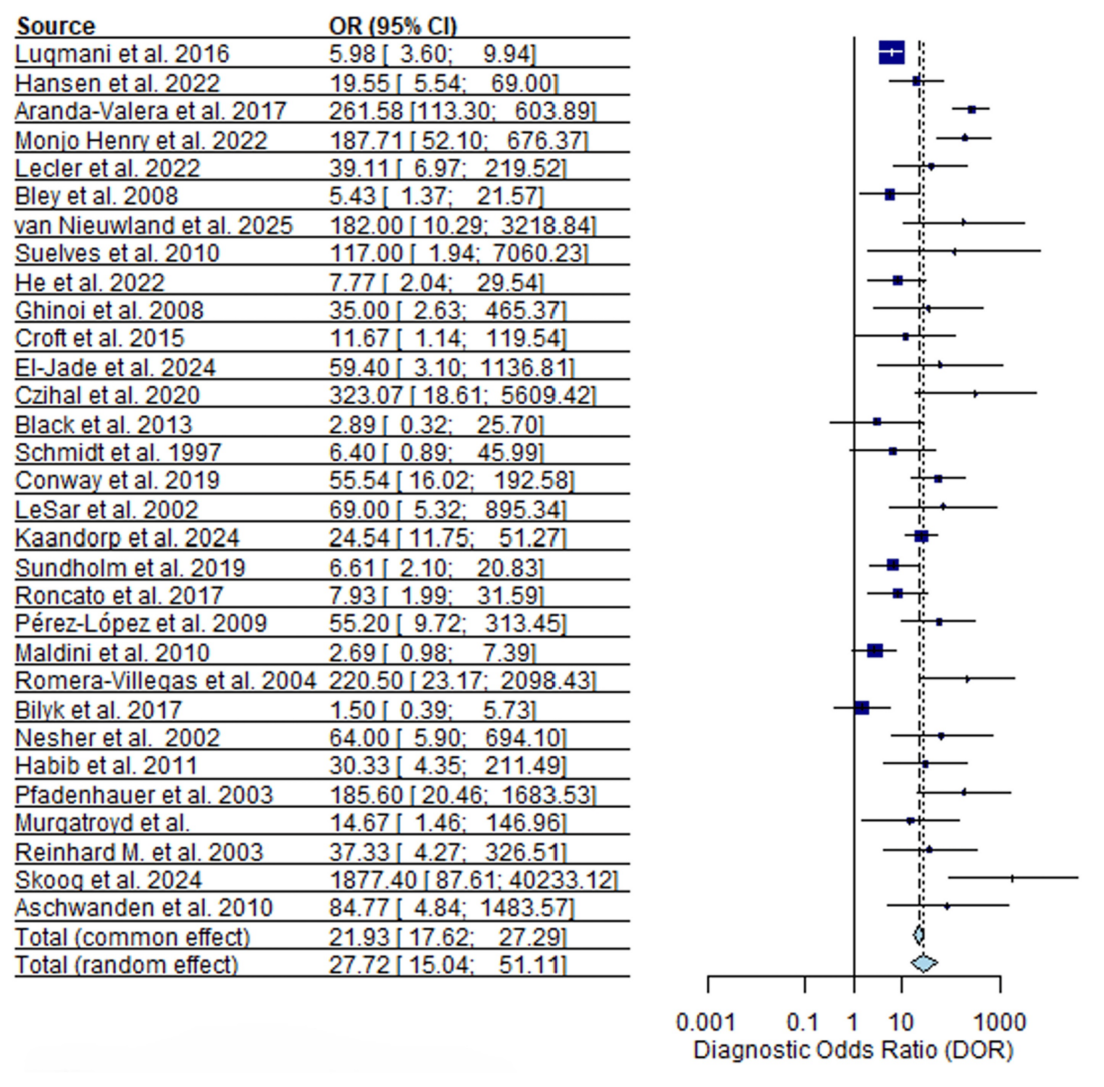
Ultrasound diagnostic odds ratios (DOR) forest plot [13-43]

Diagnostic odds ratios (DORs) for ultrasound demonstrated marked dispersion. The median DOR was 24.9 (95% CI), with individual study estimates ranging from 1.6 to 1,877 (Figure 6), indicating substantial between-study variability.

**Figure 7:**
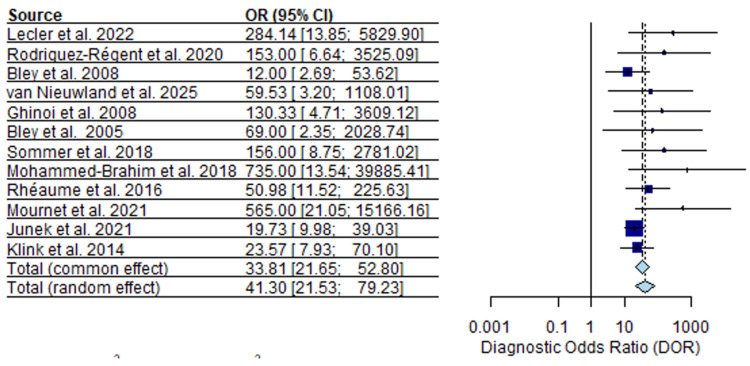
MRI diagnostic odds ratios (DOR) forest plot [17-19], [22], [44-51].

MRI DORs were higher overall than those observed for ultrasound, with a median DOR of 72.0 (95% CI) and a range from 10.4 to 735 (Figure 7).

Compared with ultrasound, MRI DOR estimates showed less dispersion across studies.

**Figure 8:**
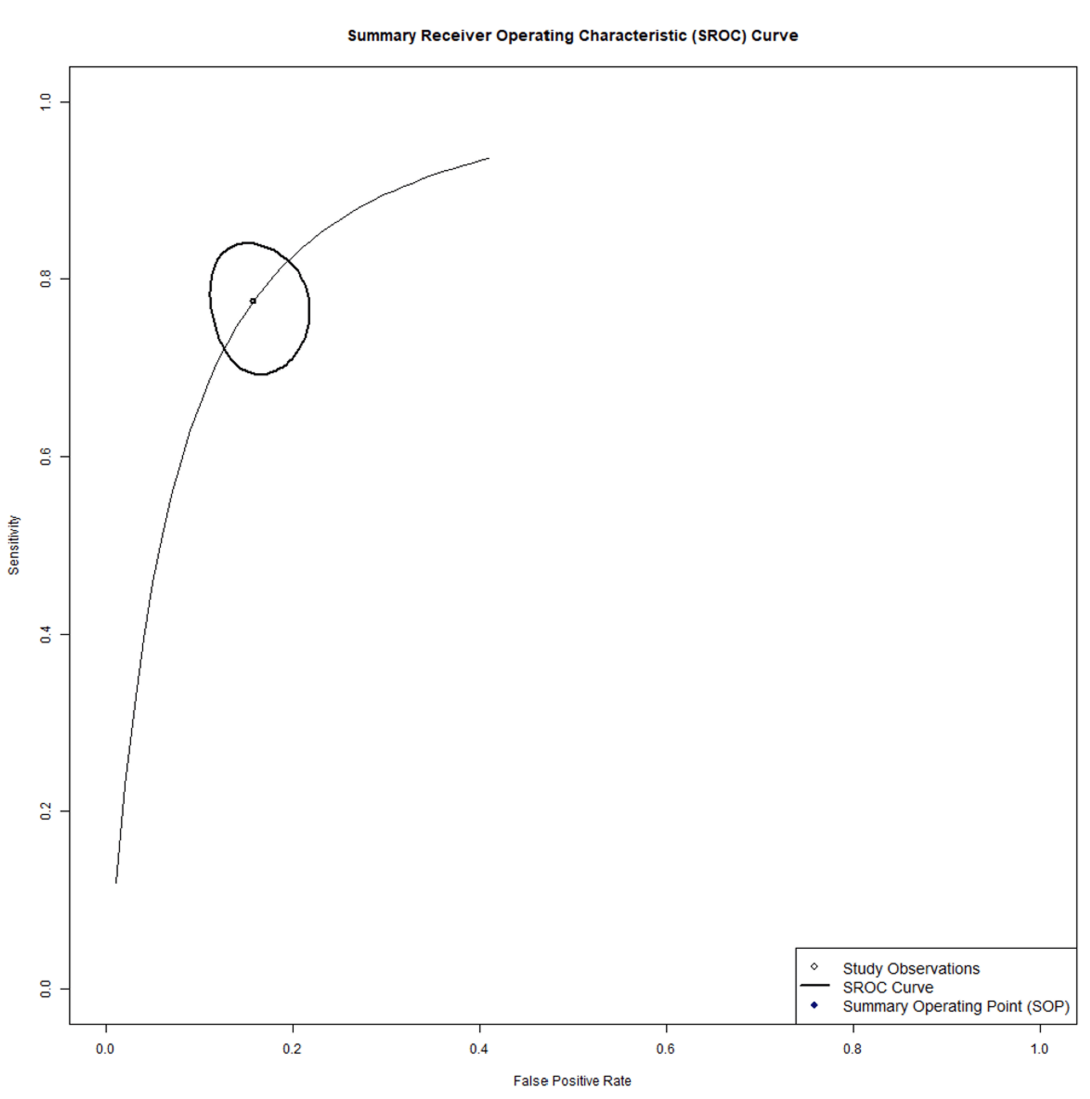
Ultrasound summary receiver operating characteristic (SROC) [13-43]

The ultrasound SROC curve showed a median operating point of 83% sensitivity and 88% specificity, with wide scatter of individual study estimates (Figure 8). 

**Figure 9:**
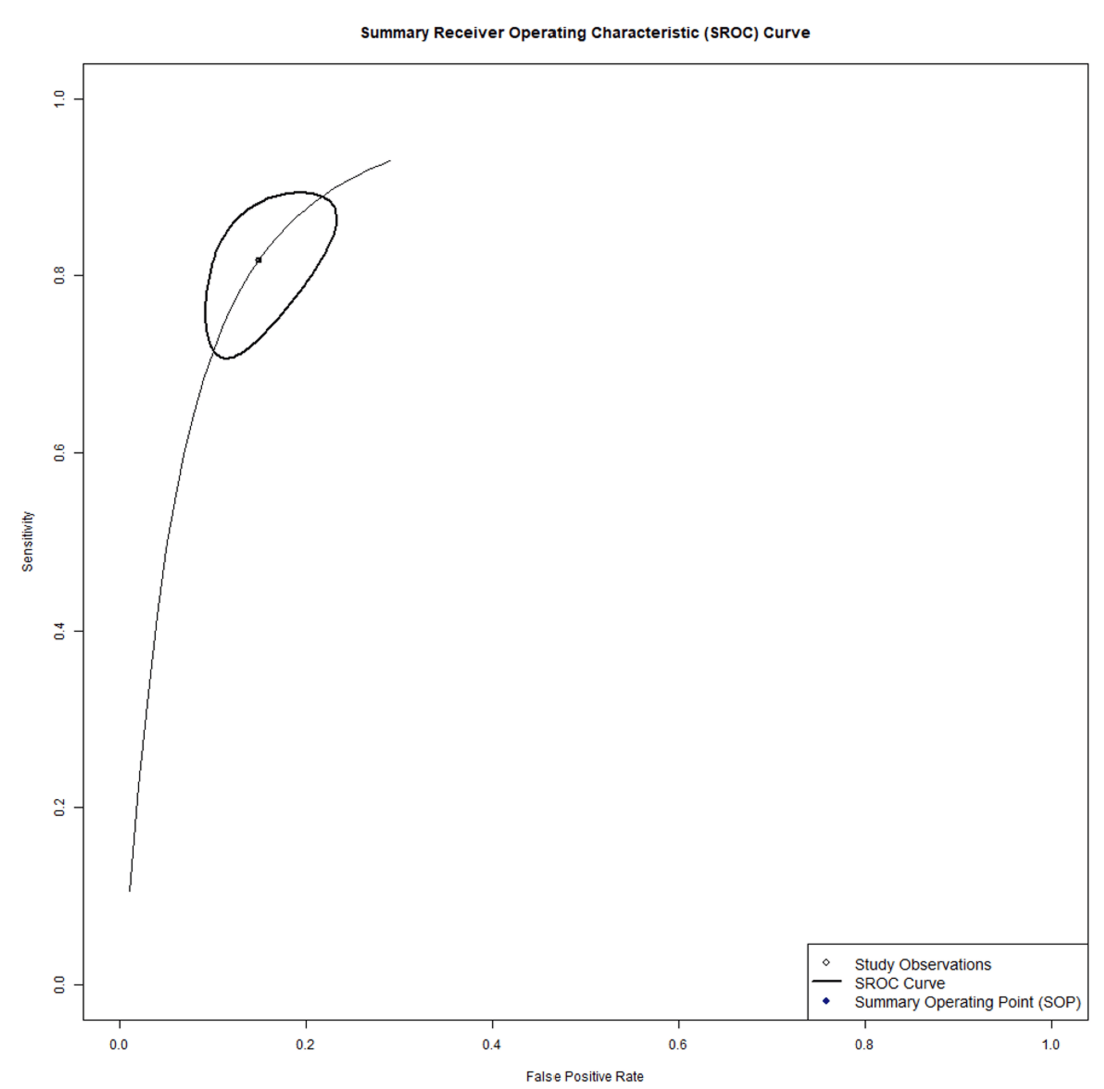
MRI summary receiver operating characteristic (SROC) [17-19], [22], [44-51].

The MRI SROC curve demonstrated a median operating point of 88% sensitivity and 96% specificity, with tighter clustering of contributing study estimates relative to ultrasound (Figure 9).

**Figure 10:**
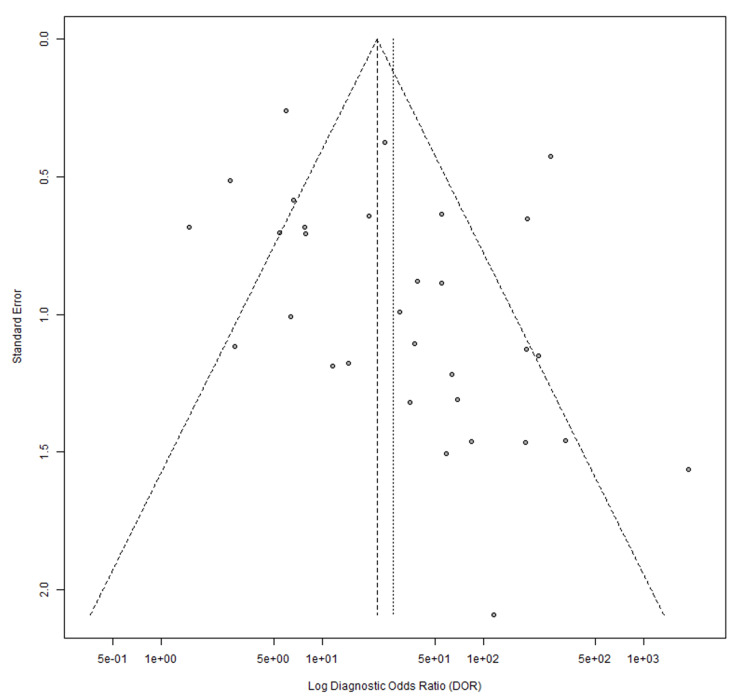
Ultrasound funnel plot [13-43]

**Figure 11:**
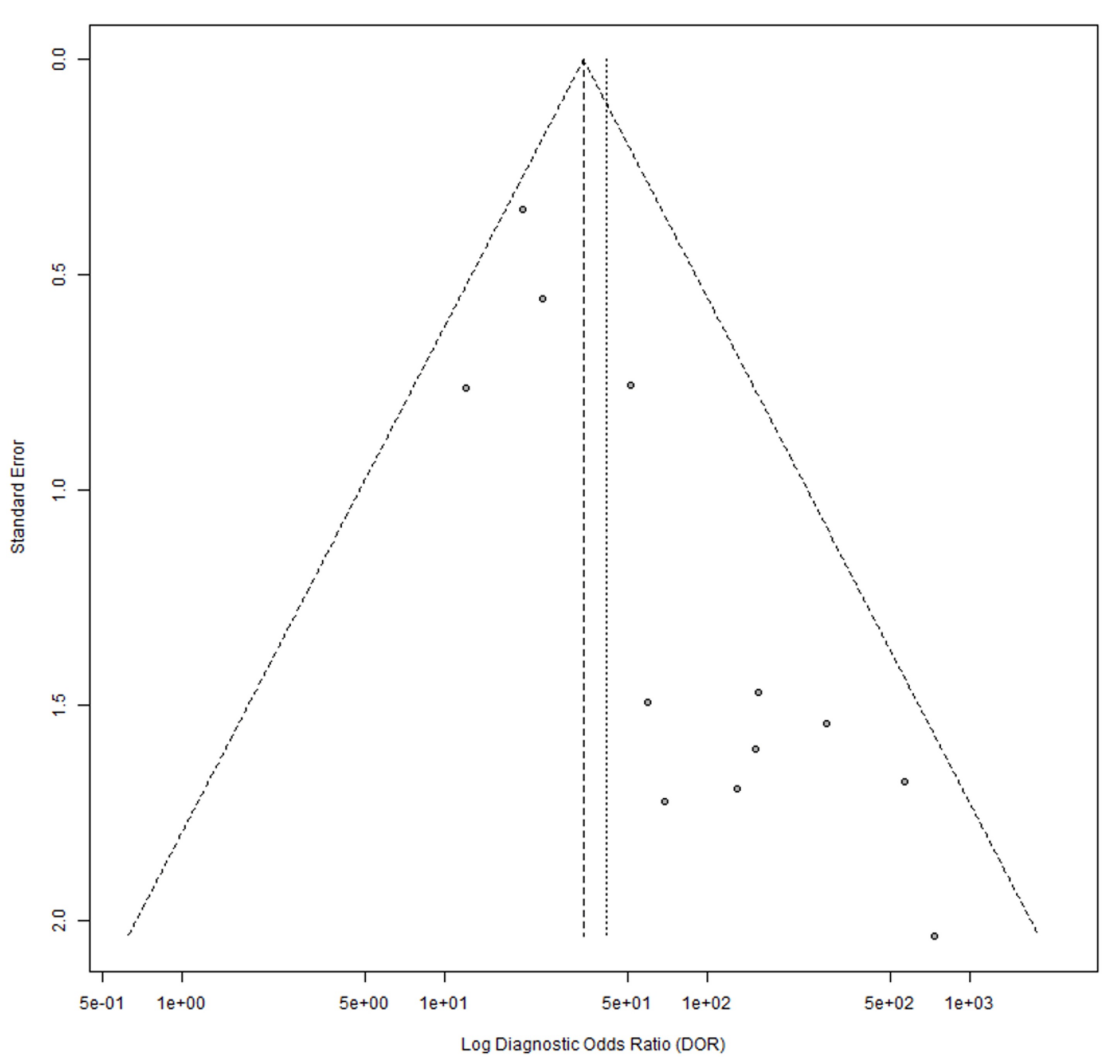
MRI funnel plot [17-19], [22], [44-51].

Publication bias was assessed using Egger’s regression test. For ultrasound studies, the test indicated borderline asymmetry (p=0.0554; bias estimate 1.5460). MRI studies demonstrated significant asymmetry (p=0.0004; bias estimate 1.5974). Funnel plots illustrating these findings are shown in Figures 10, 11.

**Figure 12:**
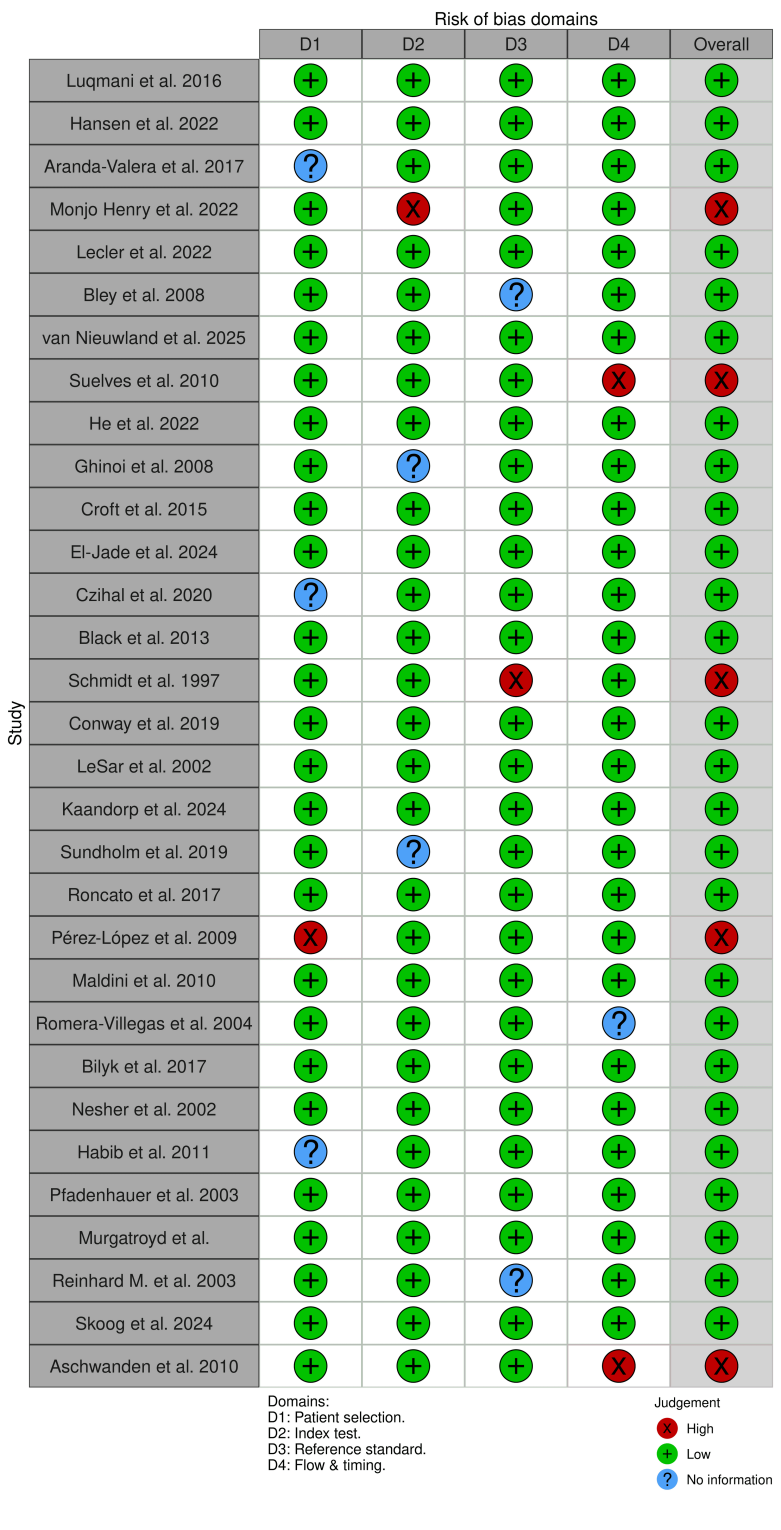
Ultrasound cohort QUADAS-2 results QUADAS 2: Quality Assessment of Diagnostic Accuracy Studies 2 tool. [13-43]

**Figure 13:**
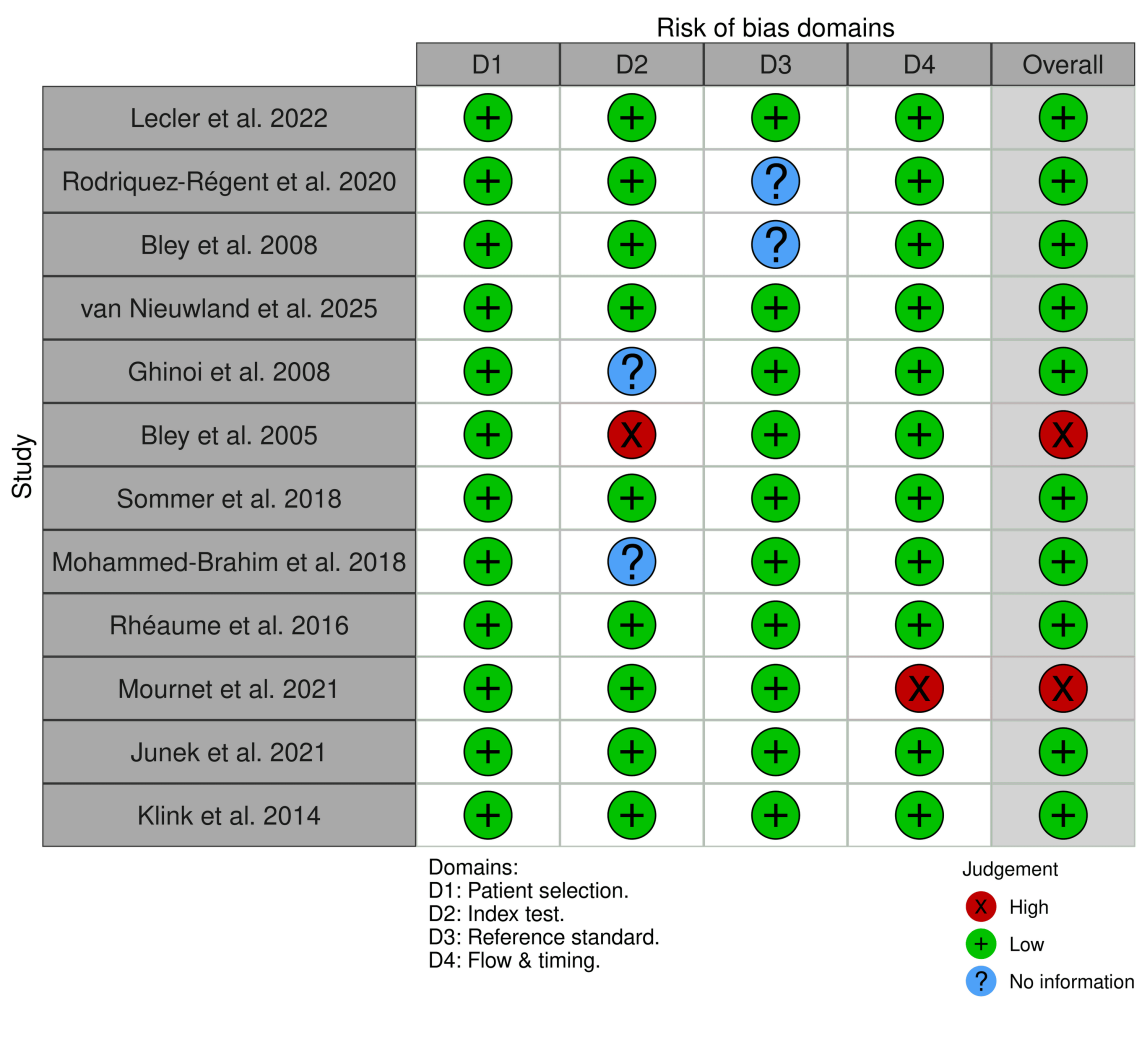
MRI cohort QUADAS-2 results QUADAS-2: Quality Assessment of Diagnostic Accuracy Studies 2 tool. [17-19], [22], [44-51].

Methodological quality was evaluated using the QUADAS-2 tool, with domain-level results presented in Figures 12, 13.

For ultrasound studies, most domains were rated as low risk of bias, though uncertainties were noted in patient selection and index test blinding in several retrospective or older studies. MRI studies showed fewer concerns in applicability domains, though the smaller number of studies limits the robustness of these assessments. Overall, QUADAS-2 findings indicate variable risk-of-bias profiles across studies rather than uniformly low risk.

**Figure 14:**
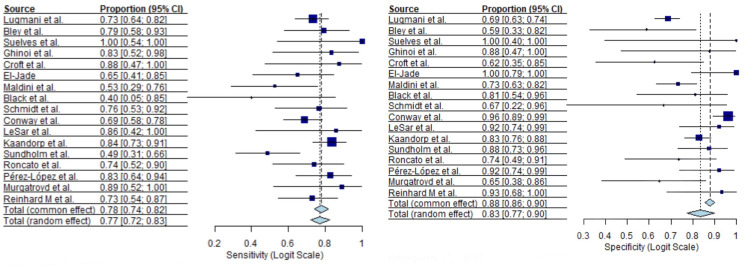
Pooled sensitivity and specificity of ultrasound in studies using temporal artery biopsy as the reference standard [13,18,20,22-24,26-34,40,41]

In the subgroup restricted to studies using temporal artery biopsy (TAB) as the reference standard, both ultrasound and MRI demonstrated consistently high diagnostic performance, with narrower confidence intervals compared with the overall analysis. For ultrasound (Figure 14), the TAB-based subgroup analysis yielded a pooled sensitivity of 0.7 (95% CI 0.72-0.83) using a random-effects model and 0.78 (95% CI 0.74-0.82) under common effects.

Pooled specificity was 0.83 (95% CI 0.77-0.90) with random effects and 0.88 (95% CI 0.86-0.90) with common effects. While several ultrasound studies reported perfect or near-perfect specificity, sensitivity estimates were more variable across studies, with some reporting values below 0.60.

**Figure 15:**
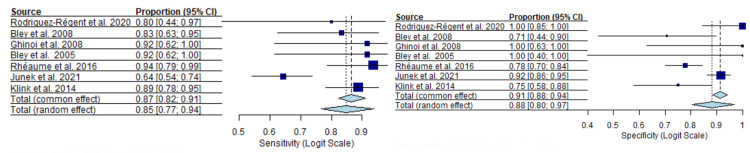
Pooled sensitivity and specificity of MRI in studies using temporal artery biopsy as the reference standard [18,22,44,45,48,50,51]

For MRI (Figure 15), pooled sensitivity was 0.85 (95% CI 0.77-0.94) using a random-effects model and 0.87 (95% CI 0.82-0.91) under a common-effects model.

Pooled specificity for MRI was similarly high, reaching 0.88 (95% CI 0.80-0.97) with random effects and 0.91 (95% CI 0.88-0.94) with common effects. Individual study estimates showed moderate variability, particularly for sensitivity, although most point estimates clustered above 0.75.

Discussion

This meta-analysis indicates that high-resolution MRI of the cranial arteries is reported with greater consistency and less between-study variability than CDUS for diagnosing GCA. Reported CDUS accuracy is heterogeneous: a unilateral halo sign has been associated with pooled sensitivity of about 68% and specificity of 91%, while bilateral halo signs produced lower pooled sensitivity (43%) but very high specificity (100%), accompanied by substantial heterogeneity across studies [52]. In our pooled DOR analysis, CDUS values ranged widely (1.6-1,877), whereas MRI DORs were more tightly clustered around a higher median (72.0), a pattern that suggests more uniform discrimination by MRI in the published literature.

This apparent consistency for MRI should be interpreted with caution. MRI studies are fewer in number and are often performed in specialized academic centers using dedicated head coils and standardized vessel-wall sequences. Such settings can magnify measured performance through selection of patients, protocol standardization, and possibly publication bias. Thus, some of the observed difference may reflect study context and methodology rather than intrinsic superiority of the modality. Conversely, CDUS performance is highly sensitive to operator experience, choice of probe and Doppler settings, and the completeness of the arterial survey (temporal and axillary arteries in particular). Multicenter evidence shows that ultrasound accuracy improves in expert hands and with standardized protocols, but falls in less-experienced environments [53-60]. Importantly, CDUS can achieve high accuracy when performed by trained sonographers who follow guideline-recommended techniques. MRI interpretation is not free from similar concerns. Accurate MRI assessment requires radiologists experienced in vessel-wall imaging and appropriate pulse sequences; results can be affected by scanner field strength, sequence selection, spatial resolution, and timing of contrast administration. These MRI-specific sources of variability are less well reported in the literature but are relevant for implementation outside specialized centers. Taken together, both modalities have factors that influence real-world performance: CDUS is more operator-dependent, while MRI performance depends on scanner and protocol characteristics and reader expertise.

In our pooled data, forest plots of DOR illustrated this contrast. Ultrasound DORs ranged from 1.6 to 1,877, reflecting extreme heterogeneity (Figure 6), whereas MRI DORs clustered around a higher median value (72.0). This suggests that, within published studies, MRI offers more stable discrimination between GCA-positive and GCA-negative patients across centers. However, ultrasound accuracy can be high in expert hands and may decline in settings with less experience. This interpretation aligns with multicenter evidence showing that vascular ultrasound performance in suspected GCA varies according to operator expertise, protocols, and arterial territories examined, with more consistent results reported in specialized centers [53]. In TAB-confirmed studies, both ultrasound and MRI showed high diagnostic performance with reduced heterogeneity, providing more precise estimates than the overall analysis. MRI demonstrated slightly higher pooled sensitivity and specificity, though overlapping confidence intervals indicate these differences are modest and study-level rather than within-patient. MRI sensitivity was more variable, likely reflecting differences in imaging sequences, scanner resolution, timing relative to treatment, and interpretation criteria. 

These findings have practical implications. The wide variability in CDUS performance likely reflects its operator dependence and sensitivity to technical factors. Diagnostic accuracy depends on sonographer experience, equipment, and scanning protocol [53-59]. Use of high-frequency linear probes, optimization of Doppler settings, and a standardized approach to examining temporal and axillary arteries reduce false-negative and false-positive findings [54]. Even with good technique, reported CDUS sensitivity varies widely: one meta-analysis found that requiring halo signs in more than one artery increased sensitivity toward 100%, while many single-center series reported substantially lower values [53]. MRI, by contrast, relies on standardized pulse sequences and dedicated head coils to depict mural inflammation, which may contribute to more uniform results in controlled settings. For example, one study reported similar sensitivity for MRI and CDUS in detecting temporal artery inflammation (60% vs 76%), but MRI achieved 100% specificity [58]. Clinically, this means that a negative ultrasound; particularly outside expert centers, may not exclude GCA, while a positive MRI strongly supports the diagnosis, acknowledging that MRI performance may vary with local expertise and protocols. Current EULAR and ACR guidance favors early ultrasound; including assessment of axillary arteries, within fast-track pathways because of its accessibility and speed. Our pooled results support ultrasound as a pragmatic first-line test in many settings but emphasize that a negative or equivocal ultrasound, particularly when obtained outside experienced units, does not exclude GCA. In those circumstances, MRI can provide complementary information and may have a high positive predictive value when obtained using appropriate vessel-wall protocols. 

Timing and pre-test treatment substantially affect imaging yield. In routine practice, patients with suspected GCA are often started on high-dose corticosteroids immediately to reduce the risk of vision loss. Both ultrasound halo signs and MRI vessel-wall enhancement can diminish within days of treatment [57,58]. One review noted that ultrasound and MRI findings may normalize rapidly and should therefore be obtained as early as possible, preferably before or within the first day of treatment [58]. Guidelines similarly emphasize early imaging, recommending ultrasound (including axillary arteries) as the initial test, while noting that delayed imaging of any type increases false-negative rates [60]. Although our meta-analysis excluded studies with prolonged steroid exposure, real-world decision-making requires balancing treatment urgency against diagnostic yield. In many fast-track pathways, ultrasound is performed at presentation because of its immediate availability [53]. When ultrasound is negative or inconclusive but clinical suspicion remains high, MRI can be considered, particularly if steroid exposure has been brief. Consequently, it remains uncertain whether MRI and CDUS are equally susceptible to early corticosteroid effects in aggregated analyses. This limitation affects how pooled estimates translate to clinical practice, where treatment is often started immediately to protect vision.

Overall, our data show that MRI is associated with more consistent performance in published studies and that CDUS accuracy is highly variable but can be excellent in experienced hands. Differences in reported performance likely reflect a combination of modality characteristics, study selection, protocol standardization, operator and reader expertise, and publication patterns. A cautious interpretive approach that references guideline frameworks and local diagnostic resources will best serve clinicians faced with suspected GCA.

Limitations

The present analysis has several limitations. Heterogeneity in imaging protocols and diagnostic thresholds; including probe frequency, Doppler settings, MRI sequences, coil use, and arterial territories examined, contributed to between-study variance and limits direct comparability. Reference standards also varied, with some studies relying on clinical diagnosis or composite outcomes rather than biopsy or long-term adjudication, introducing potential misclassification bias. The timing of corticosteroid exposure was inconsistently reported, preventing stratification of diagnostic accuracy by treatment interval. MRI studies were predominantly conducted in specialized academic centers with standardized protocols, which may overestimate performance in routine practice and increase susceptibility to publication bias. Operator and reader effects further influence accuracy, as ultrasound depends on sonographer expertise and MRI on scanner characteristics and radiologist experience, yet these factors were inconsistently reported. Finally, potential publication and selective-reporting biases could inflate pooled estimates for either modality. 

## Conclusions

In conclusion, within the published literature, MRI shows more consistent and reproducible diagnostic performance for GCA, while CDUS demonstrates greater variability related to operator dependence. Both modalities play complementary roles. Ultrasound remains an effective and scalable first-line test in experienced centers, particularly within fast-track pathways, while MRI provides useful confirmatory information when ultrasound is inconclusive or expertise is limited. Imaging choice should be guided by clinical context, local expertise, access, and timing of corticosteroid therapy. Overall, our findings support imaging-based diagnostic pathways for GCA, with careful consideration of the strengths and limitations of each modality.

## References

[1] Weyand CM, Goronzy JJ (2014). Giant-cell arteritis and polymyalgia rheumatica. N Engl J Med.

[2] Salvarani C, Cantini F, Hunder GG (2008). Polymyalgia rheumatica and giant-cell arteritis. Lancet.

[3] Klein RG, Campbell RJ, Hunder GG, Carney JA (1976). Skip lesions in temporal arteritis. Mayo Clin Proc.

[4] Ashton-Key MR, Gallagher PJ (1992). False-negative temporal artery biopsy. Am J Surg Pathol.

[5] Schmidt WA, Kraft HE, Vorpahl K, Völker L, Gromnica-Ihle EJ (1997). Color duplex ultrasonography in the diagnosis of temporal arteritis. N Engl J Med.

[6] Ball EL, Walsh SR, Tang TY, Gohil R, Clarke JM (2010). Role of ultrasonography in the diagnosis of temporal arteritis. Br J Surg.

[7] Adhithyan R, Kesav P, Thomas B, Sylaja PN, Kesavadas C (2018). High-resolution magnetic resonance vessel wall imaging in cerebrovascular diseases. Neurol India.

[8] Dejaco C, Ramiro S, Duftner C (2018). EULAR recommendations for the use of imaging in large vessel vasculitis in clinical practice. Ann Rheum Dis.

[9] Ponte C, Grayson PC, Robson JC (2022). 2022 American College of Rheumatology/EULAR classification criteria for giant cell arteritis. Arthritis Rheumatol.

[10] Whiting PF, Rutjes AW, Westwood ME (2011). QUADAS-2: a revised tool for the quality assessment of diagnostic accuracy studies. Ann Intern Med.

[11] Reitsma JB, Glas AS, Rutjes AW, Scholten RJ, Bossuyt PM, Zwinderman AH (2005). Bivariate analysis of sensitivity and specificity produces informative summary measures in diagnostic reviews. J Clin Epidemiol.

[12] McInnes MD, Moher D, Thombs BD (2018). Preferred reporting items for a systematic review and meta-analysis of diagnostic test accuracy studies: the PRISMA-DTA statement. JAMA.

[13] Luqmani R, Lee E, Singh S (2016). The role of ultrasound compared to biopsy of temporal arteries in the diagnosis and treatment of giant cell arteritis (TABUL): a diagnostic accuracy and cost-effectiveness study. Health Technol Assess.

[14] Hansen MS, Terslev L, Jensen MR (2023). Comparison of temporal artery ultrasound versus biopsy in the diagnosis of giant cell arteritis. Eye (Lond).

[15] Aranda-Valera IC, García Carazo S, Monjo Henry I, De Miguel Mendieta E (2017). Diagnostic validity of Doppler ultrasound in giant cell arteritis. Clin Exp Rheumatol.

[16] Molina-Collada J, Castrejón I, Monjo I, Fernández-Fernández E, Torres Ortiz G, Álvaro-Gracia JM, de Miguel E (2023). Performance of the 2022 ACR/EULAR giant cell arteritis classification criteria for diagnosis in patients with suspected giant cell arteritis in routine clinical care. RMD Open.

[17] Lecler A, Hage R, Charbonneau F (2022). Validation of a multimodal algorithm for diagnosing giant cell arteritis with imaging. Diagn Interv Imaging.

[18] Bley TA, Reinhard M, Hauenstein C (2008). Comparison of duplex sonography and high-resolution magnetic resonance imaging in the diagnosis of giant cell (temporal) arteritis. Arthritis Rheum.

[19] van Nieuwland M, Nienhuis PH, Haagsma C (2025). An in-depth comparison of vascular inflammation on ultrasound, FDG-PET/CT and MRI in patients with suspected giant cell arteritis. Eur J Nucl Med Mol Imaging.

[20] Suelves AM, España-Gregori E, Tembl J, Rohrweck S, Millán JM, Díaz-Llopis M (2010). Doppler ultrasound and giant cell arteritis. Clin Ophthalmol.

[21] He J, Williamson L, Ng B (2022). The diagnostic accuracy of temporal artery ultrasound and temporal artery biopsy in giant cell arteritis: a single center Australian experience over 10 years. Int J Rheum Dis.

[22] Ghinoi A, Zuccoli G, Nicolini A (2008). 1T magnetic resonance imaging in the diagnosis of giant cell arteritis: comparison with ultrasonography and physical examination of temporal arteries. Clin Exp Rheumatol.

[23] Croft AP, Thompson N, Duddy MJ, Barton C, Khattak F, Mollan SP, Jobanputra P (2015). Cranial ultrasound for the diagnosis of giant cell arteritis. A retrospective cohort study. J R Coll Physicians Edinb.

[24] El-Jade M (2024). The role of color doppler ultrasonography in the diagnosis of giant cell arteritis in ophthalmic patients. J Ultrasound.

[25] Czihal M, Köhler A, Lottspeich C (2021). Temporal artery compression sonography for the diagnosis of giant cell arteritis in elderly patients with acute ocular arterial occlusions. Rheumatology (Oxford).

[26] Black R, Roach D, Rischmueller M, Lester SL, Hill CL (2013). The use of temporal artery ultrasound in the diagnosis of giant cell arteritis in routine practice. Int J Rheum Dis.

[27] Schmid R, Hermann M, Yannar A, Baumgartner RW (2002). Color duplex ultrasound of the temporal artery: replacement for biopsy in temporal arteritis (Article in German). Ophthalmologica.

[28] Conway R, O'Neill L, McCarthy GM (2019). Performance characteristics and predictors of temporal artery ultrasound for the diagnosis of giant cell arteritis in routine clinical practice in a prospective cohort. Clin Exp Rheumatol.

[29] LeSar CJ, Meier GH, DeMasi RJ (2002). The utility of color duplex ultrasonography in the diagnosis of temporal arteritis. J Vasc Surg.

[30] Kaandorp BI, Raterman HG, Stam F, Gamala M, Meijer-Jorna LB, Kalb FB, Wallis JW (2024). Determination of the value of color Doppler ultrasound in patients with a clinical suspicion of giant cell arteritis. ACR Open Rheumatol.

[31] Sundholm JK, Pettersson T, Paetau A, Albäck A, Sarkola T (2019). Diagnostic performance and utility of very high-resolution ultrasonography in diagnosing giant cell arteritis of the temporal artery. Rheumatol Adv Pract.

[32] Roncato C, Allix-Béguec C, Brottier-Mancini E, Gombert B, Denis G (2017). Diagnostic performance of colour duplex ultrasonography along with temporal artery biopsy in suspicion of giant cell arteritis. Clin Exp Rheumatol.

[33] Pérez López J, Solans Laqué R, Bosch Gil JA, Molina Cateriano C, Huguet Redecilla P, Vilardell Tarrés M (2009). Colour-duplex ultrasonography of the temporal and ophthalmic arteries in the diagnosis and follow-up of giant cell arteritis. Clin Exp Rheumatol.

[34] Maldini C, Dépinay-Dhellemmes C, Tra TT (2010). Limited value of temporal artery ultrasonography examinations for diagnosis of giant cell arteritis: analysis of 77 subjects. J Rheumatol.

[35] Romera-Villegas A, Vila-Coll R, Poca-Dias V, Cairols-Castellote MA (2004). The role of color duplex sonography in the diagnosis of giant cell arteritis. J Ultrasound Med.

[36] Bilyk JR, Murchison AP, Leiby BT, Sergott RC, Eagle RC, Needleman L, Savino PJ (2017). The utility of color duplex ultrasonography in the diagnosis of giant cell arteritis: a prospective, masked study. (An American Ophthalmological Society thesis). Trans Am Ophthalmol Soc.

[37] Nesher G, Shemesh D, Mates M, Sonnenblick M, Abramowitz HB (2002). The predictive value of the halo sign in color Doppler ultrasonography of the temporal arteries for diagnosing giant cell arteritis. J Rheumatol.

[38] Habib HM, Essa AA, Hassan AA (2012). Color duplex ultrasonography of temporal arteries: role in diagnosis and follow-up of suspected cases of temporal arteritis. Clin Rheumatol.

[39] Pfadenhauer K, Weber H (2003). Giant cell arteritis of the occipital arteries-a prospective color coded duplex sonography study in 78 patients. J Neurol.

[40] Murgatroyd H, Nimmo M, Evans A, MacEwen C (2003). The use of ultrasound as an aid in the diagnosis of giant cell arteritis: a pilot study comparing histological features with ultrasound findings. Eye (Lond).

[41] Reinhard M, Schmidt D, Hetzel A (2004). Color-coded sonography in suspected temporal arteritis-experiences after 83 cases. Rheumatol Int.

[42] Skoog J, Svensson C, Eriksson P, Sjöwall C, Zachrisson H (2024). High-frequency ultrasound with superb microvascular imaging: a potential tool for ultrasound assessment in patients with giant cell arteritis. Front Med (Lausanne).

[43] Aschwanden M, Kesten F, Stern M (2010). Vascular involvement in patients with giant cell arteritis determined by duplex sonography of 2x11 arterial regions. Ann Rheum Dis.

[44] Rodriguez-Régent C, Ben Hassen W, Seners P, Oppenheim C, Régent A (2020). 3D T1-weighted black-blood magnetic resonance imaging for the diagnosis of giant cell arteritis. Clin Exp Rheumatol.

[45] Bley TA, Wieben O, Uhl M, Thiel J, Schmidt D, Langer M (2005). High-resolution MRI in giant cell arteritis: imaging of the wall of the superficial temporal artery. AJR Am J Roentgenol.

[46] Sommer NN, Treitl KM, Coppenrath E (2018). Three-dimensional high-resolution black-blood magnetic resonance imaging for detection of arteritic anterior ischemic optic neuropathy in patients with giant cell arteritis. Invest Radiol.

[47] Mohammed-Brahim N, Clavel G, Charbonneau F (2019). Three Tesla 3D high-resolution vessel wall MRI of the orbit may differentiate arteritic from nonarteritic anterior ischemic optic neuropathy. Invest Radiol.

[48] Boulon C, Skopinski S, Constans J (2017). High-resolution magnetic resonance imaging of scalp arteries and ultrasound of temporal arteries: a winning strategy for diagnosing giant cell arteritis? Comment on the article by Rhéaume et al. Arthritis Rheumatol.

[49] Mournet S, Sené T, Charbonneau F (2021). High-resolution MRI demonstrates signal abnormalities of the 3rd cranial nerve in giant cell arteritis patients with 3rd cranial nerve impairment. Eur Radiol.

[50] Junek M, Hu A, Garner S, Rebello R, Legault K, Beattie K, Khalidi N (2021). Contextualizing temporal arterial magnetic resonance angiography in the diagnosis of giant cell arteritis: a retrospective cohort study. Rheumatology (Oxford).

[51] Klink T, Geiger J, Both M (2014). Giant cell arteritis: diagnostic accuracy of MR imaging of superficial cranial arteries in initial diagnosis-results from a multicenter trial. Radiology.

[52] Arida A, Kyprianou M, Kanakis M, Sfikakis PP (2010). The diagnostic value of ultrasonography-derived edema of the temporal artery wall in giant cell arteritis: a second meta-analysis. BMC Musculoskelet Disord.

[53] van der Geest KS, Borg F, Kayani A (2020). Novel ultrasonographic Halo score for giant cell arteritis: assessment of diagnostic accuracy and association with ocular ischaemia. Ann Rheum Dis.

[54] Chrysidis S, Duftner C, Dejaco C (2018). Definitions and reliability assessment of elementary ultrasound lesions in giant cell arteritis: a study from the OMERACT large vessel vasculitis ultrasound working group. RMD Open.

[55] Dejaco C, Ramiro S, Bond M (2024). EULAR recommendations for the use of imaging in large vessel vasculitis in clinical practice: 2023 update. Ann Rheum Dis.

[56] Hauenstein C, Reinhard M, Geiger J (2012). Effects of early corticosteroid treatment on magnetic resonance imaging and ultrasonography findings in giant cell arteritis. Rheumatology (Oxford).

[57] Duftner C, Dejaco C, Sepriano A, Falzon L, Schmidt WA, Ramiro S (2018). Imaging in diagnosis, outcome prediction and monitoring of large vessel vasculitis: a systematic literature review and meta-analysis informing the EULAR recommendations. RMD Open.

[58] Ponte C, Martins-Martinho J, Luqmani RA (2020). Diagnosis of giant cell arteritis. Rheumatology (Oxford).

[59] Mackie SL, Dejaco C, Appenzeller S (2020). British Society for Rheumatology guideline on diagnosis and treatment of giant cell arteritis. Rheumatology (Oxford).

[60] Schäfer VS, Juche A, Ramiro S, Krause A, Schmidt WA (2017). Ultrasound cut-off values for intima-media thickness of temporal, facial and axillary arteries in giant cell arteritis. Rheumatology (Oxford).

